# Comparison of the Freiburg and Charlson Comorbidity Indices in Predicting Overall Survival in Elderly Patients with Newly Diagnosed Multiple Myeloma

**DOI:** 10.1155/2014/437852

**Published:** 2014-07-10

**Authors:** Sung Min Kim, Moon Jin Kim, Hyun Ae Jung, Kihyun Kim, Seok Jin Kim, Jun Ho Jang, Won Seog Kim, Chul Won Jung

**Affiliations:** Division of Hematology-Oncology, Department of Medicine, Samsung Medical Center, School of Medicine, Sungkyunkwan University, 81 Irwon-ro, Gangnam-gu, Seoul 135-710, Republic of Korea

## Abstract

Multiple myeloma occurs primarily in elderly patients. Considering the high prevalence of comorbidities, comorbidity is an important issue for the management of myeloma. However, the impact of comorbidity on clinical outcomes has not been fully investigated. We retrospectively analyzed patients with newly diagnosed myeloma. Comorbidities were assessed based on the Charlson comorbidity index (CCI) and the Freiburg comorbidity index (FCI). The CCI is a summary measure of 19 comorbid conditions. FCI is determined by performance status, renal impairment, and lung disease. This study included 127 patients with a median age of 71 years. Approximately half of the patients had additional disorders at the time of diagnosis, and diabetes mellitus was the most frequent diagnosis (18.9%). The most significant factors for prognosis among patient-related conditions were a history of solid cancer and performance status (ECOG ≥ 2). The FCI score was divided into 3 groups (0, 1, and 2-3), and the CCI score was divided into 2 groups (2-3 and ≥4). FCI was a strong prognostic tool for OS (*P* > 0.001) and predicted clinical outcome better than CCI (*P* = 0.059). In conclusion, FCI was more useful than CCI in predicting overall survival in elderly patients with myeloma.

## 1. Introduction

Multiple myeloma (MM) is a hematologic malignancy of plasma cells that results in bone destruction, marrow failure, and renal impairment. The median age at the time of diagnosis is 70 years, with 36% of patients younger than 65 years, 27% aged 65 to 74 years, and 37% older than 75 years [1]. Considering the increasing life expectancy of the general population, the number of geriatric patients affected by MM is expected to increase over time.

Risk stratification of myeloma using the international staging system (ISS) and host factors such as age, performance status, and comorbidities are thought to be important for determining prognosis and choosing treatment options [2–4]. In 2011, Palumbo et al. suggested that appropriate screening for age (>75 years) and vulnerability, in addition to assessment of cardiac, pulmonary, renal, hepatic, and neurological functions, at the start of therapy allows treatment strategies to be individualized and drug doses to be tailored to improve tolerability and optimize efficacy [5]. Their study emphasized that elderly MM patients are more susceptible to side effects and are often unable to tolerate full drug doses. For these patients, lower-dose intensity regimens improve the safety profile and thus optimize the treatment outcome. However, this study was not based specifically on MM because there were few data on the impact of vulnerability on outcomes in MM patients [5]. Nonetheless, in general, several studies have reported problems related to comorbidity and cancer treatment in elderly patients [6–10].

Kleber et al. developed the Freiburg comorbidity index (FCI) to assess patient-related conditions as a risk factor for MM. The FCI is composed of three comorbidity factors: renal impairment, moderate to severe lung disease, and performance status. Interestingly, the FCI showed strong clinical relevance for overall survival (OS) and progression-free survival (PFS). Moreover, compared with other comorbidity indices, such as the Charlson comorbidity index (CCI), hematopoietic cell transplantation-specific comorbidity index (HCT-CI), Kaplan-Feinstein (KF), and Satariano index (SI), FCI was better able to stratify risk in patients with MM [11].

Although CCI is a widely used tool for assessing comorbidity in malignancy, this comorbidity index is complicated and difficult to apply. Moreover, there is no proven cut-off value that divides patients into low- or high-risk groups. As a result, several studies have determined their own cut-off values [11–16]. For example, Offidani et al. suggested a vulnerability score consisting of performance status and comorbidity score of CCI 0 or ≥1 [15].

Because of the increased incidence of multiple myeloma with aging and the fact that elderly patients have more comorbidity than younger patients, in the present study, we assessed comorbidities at diagnosis, the impact of host factors on OS, and compared CCI and FCI as prognostic factors in newly diagnosed elderly patients with MM.

## 2. Methods

### 2.1. Study Design

This study was a retrospective, single-center case series. OS was calculated as the time from diagnosis to death from any cause. Adverse events were graded according to the National Cancer Institute Common Toxicity Criteria (NCI-CTC) version 4.0. This study was reviewed and approved by the Institutional Review Board.

### 2.2. Patients

A total of 127 consecutive patients aged 65 years and older who were newly diagnosed with symptomatic MM at the Samsung Medical Center, Seoul, South Korea, between January 1, 1999, and June 30, 2011, met the inclusion criteria for this study. We excluded patients with amyloidosis, those who were suitable for autologous stem cell transplantation (ASCT), and those who were lost to follow-up within 6 months from the time of diagnosis due to any cause except death. The last follow-up date was March 31, 2013.

### 2.3. Charlson Comorbidity Index (CCI)

The CCI (Table 1) is a summary measure of 19 comorbid conditions that are each weighted from 1 to 6 based on disease severity. This measure provides a total score ranging from 0 to 37 [17]. Information on comorbidity was extracted from a detailed review of each patient's medical records and laboratory values at the time of diagnosis. In the original study, MM was included in the classification of lymphoma for convenience. In this study, lymphoma was defined, as in the original study, except that the definition did not include myeloma.

In addition, according to the original study, each decade of age over 40 would add 1 point to the risk value (i.e., 50 years = +1 point), and the age point would be added to the score of the comorbidity index. In this study, we used the method described by Kleber et al., which adds the age point to the CCI score [11].

### 2.4. Freiburg Comorbidity Index (FCI)

Renal impairment is defined as estimated glomerular filtration rate (eGFR) ≤30 mL/min/1.73 m^2^, based on the modification of diet in renal disease (MDRD) study equation [18]. Poor performance status is based on a Karnofsky performance status (KPS) score ≤70. Moderate or severe lung disease is defined in the same manner as in the CCI [11]. Each of the variables contributes 1 point and the FCI is a summation of these points; thus, the FCI value ranges from 0 to 3.

### 2.5. Statistical Analysis

Numerical variables are summarized by median and range and categorical variables are described by count and relative frequency (%) of subjects in each category. Comparison of the distribution of categorical variables in the different groups was performed with either Fisher's exact test or the *χ* ^2^ test. OS was estimated using the Kaplan-Meier methodology. Uni- and multivariate Cox regression analyses were applied to assess factors affecting OS. Components with a *P* value less than 0.05 in univariate analysis were included in the subsequent multivariate analysis.

These analyses were performed using PASW statistics 18.0.0 (WinWrap, IBM, New York, USA). Null hypotheses of no difference were rejected if *P* values were less than 0.05.

## 3. Results

### 3.1. Patient Characteristics

During the study period, a total of 159 patients aged 65 years or older were newly diagnosed with symptomatic MM. Among them, 22 patients were excluded because of a combined diagnosis of amyloidosis, early follow-up loss, or ASCT. Therefore, data from 127 patients were included in the analysis.

Table 2 shows baseline characteristics of patients at the time of diagnosis. The median overall survival of all patients was 34.1 months, and the median follow-up duration for the surviving patients was 46 months. The median age of the patients was 71 years and 26.8% of the patients were aged 75 years or older. Performance status was evaluated by ECOG status. In FCI, performance status was assessed by KPS, and, by definition, ECOG grade 2 is interchangeable with KPS grade 70 [19–21].

The prevalence of comorbidity at the time of diagnosis was 48.8% (Table 3). The most frequent comorbid condition was diabetes without end organ damage (*n* = 24, 18.9%). The median CCI score, including age points, was 3 (range: 2 to 13). The CCI score was divided into two groups based on the median score; CCI scores of 2-3 were classified as the low CCI score group, and CCI scores ≥4 were classified as the high CCI score group. In this study, 53 and 74 patients belonged to the low and high CCI score groups, respectively. When using the FCI classification, 59, 54, and 12 patients belonged to the 0, 1, and 2-3 score groups, respectively.

### 3.2. Host Factors and Comorbidity Indices as Prognostic Factors

We analyzed the impact of host factors, such as age, sex, performance status, and each of the comorbidities on OS (Table 4). The results showed that all of the factors constituting FCI, such as performance status, chronic lung disease, and eGFR, were significant for OS. In addition, any tumor, metastatic solid tumor, cerebrovascular disease, and ISS each had statistical significance. When multivariate analysis was conducted with these factors, only performance status and previous cancer history (regardless of metastasis) remained significant.

**Figure 1:**
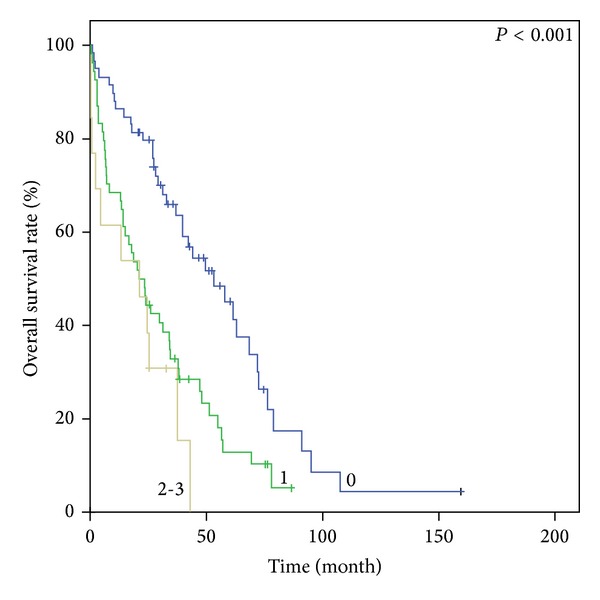
Kaplan-Meier survival curves of comorbidity index score groups. OS according to FCI.

**Figure 2:**
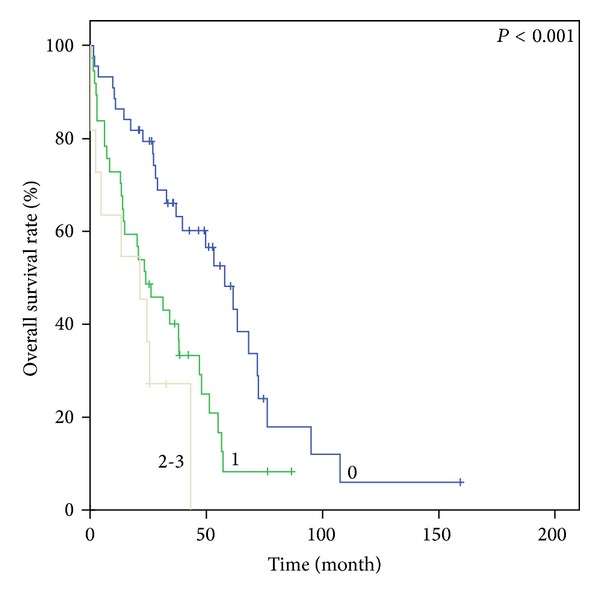
Kaplan-Meier survival curves of comorbidity index score groups. OS according to FCI in patients aged 65–74 years.

**Figure 3:**
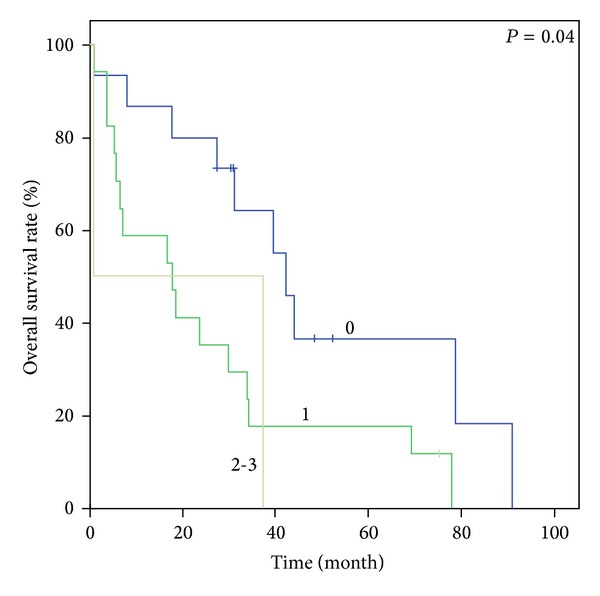
Kaplan-Meier survival curves of comorbidity index score groups. OS according to FCI in patients aged ≥75 years.

**Figure 4:**
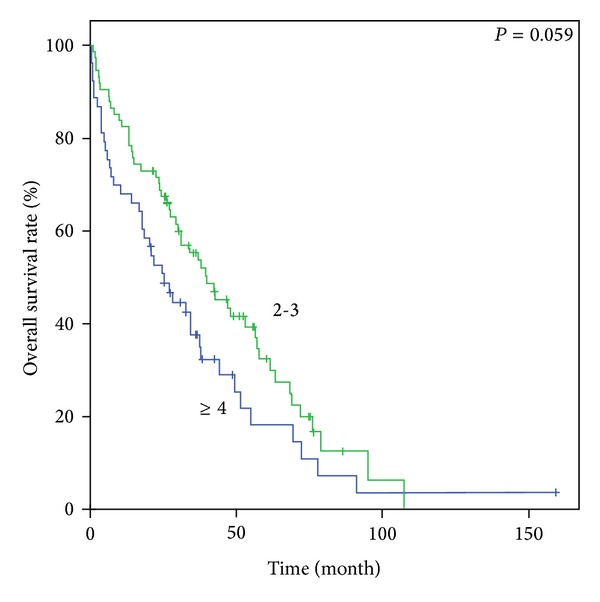
Kaplan-Meier survival curves of comorbidity index score groups. OS according to CCI.

Figures 1, 2, 3, and 4 show Kaplan-Meier survival curves for both comorbidity indices. FCI effectively predicted the OS of the three different groups (*P* < 0.001). The median survival times were 55.0 months, 29.5 months, and 19.5 months for FCI scores of 0, 1, and 2-3, respectively. Although the *P* value was not statistically significant, CCI also distinguished between the two score groups for OS (44.8 months versus 34.7 months, *P* = 0.059). However, OS based on CCI without age points did not demonstrate clinical relevance (*P* = 0.147). In contrast, FCI was significant in subgroup analysis for age groups (65–74 and ≥75 years; *P* < 0.001 and 0.04, resp.)

### 3.3. Serious Adverse Events

We defined serious adverse events as grade ≥4 for hematologic adverse events and grade ≥3 for nonhematologic adverse events, according to NCI-CTC version 4.0. The most frequent serious adverse event was infection (*n* = 35, 30.0%) followed by neutropenia and anemia (*n* = 15, 12.7% for both). Grade ≥3 nonhematologic adverse events occurred in 50% of patients, whereas grade ≥4 hematologic adverse events occurred in 22.9% of patients. Grade 5 adverse events due to any cause occurred in 6.8% of the patients, as shown in Table 5.

## 4. Discussion

This study assessed comorbidities at diagnosis of MM, the impact of host factors on overall survival, and compared CCI and FCI as prognostic factors in newly diagnosed elderly patients.

Univariate analysis revealed that performance status, ISS, and several comorbid conditions such as chronic lung disease, azotemia (eGFR < 30 mL/min/1.73 m^2^), presence of any tumor, metastatic solid tumor, and cerebrovascular disease were significant factors. However, azotemia as defined by CCI (serum creatinine ≥ 3 mg/mL) was not a prognostic factor. In multivariate analysis, azotemia, as defined by impaired eGFR or chronic lung disease, was not shown to be a significant risk factor in our study. In contrast, a history of cancer, regardless of whether metastasis occurred, was the strongest prognostic factor for elderly patients with myeloma. Unfortunately, use of novel agents over conventional drugs did not significantly improve OS, although this might reflect the relatively short period of use of novel agents.

Although two components of FCI-renal impairment and moderate or severe lung disease failed to demonstrate significance in multivariate analysis, when we compared both comorbidity indices and overall survival, the FCI showed a greater ability to separate OS among the three score groups (*P* < 0.001). The CCI score including age points was not statistically significant but was still valuable and superior to the CCI without age points. As briefly mentioned above, the CCI score without age points did not discriminate for OS.

FCI provides a clear definition of each component and all three components were statistically significant, at least in univariate analysis. In contrast, CCI is more subjective and only 4 among 19 conditions were significant. Most importantly, at the present time, CCI does not have any standard cut-off value. Various studies have divided CCI scores into groups of 0, 1-2, and ≥3; 0, 1, and ≥2; or 0 and ≥1. Some studies included an age point, but others did not [11–14]. These variations might explain why FCI is more predictable than CCI.

Moreover, FCI is also very simple to apply. FCI consists of performance status, moderate or severe lung disease, and azotemia, and each of these factors is worth 1 point. The FCI score is, therefore, a simple summation of these three factors. In contrast, CCI consists of 19 comorbid conditions, and, within the same disease, scores are weighted based on severity ranging from 1 to 6 points. In addition, an age point is calculated and added to the CCI score. Comorbidity definitions frequently use a symptomatic grade.

In this study, all 118 patients who were treated with chemotherapy received a full dose of chemotherapeutic agents as scheduled. Interestingly, the profile for serious adverse events showed that treatment was relatively safe and adverse events were easily controllable. In fact, since the approval of various novel agents, clinical outcomes such as survival and toxicity profiles have improved in transplant-ineligible elderly patients with multiple myeloma [22–27]. Thus, we propose that the full dose of chemotherapy might be tolerated, regardless of the presence of comorbid conditions, even though this is against the recommendation that chemotherapy dose reduction is required for patients 75 years or older or those with cardiac, pulmonary, hepatic, renal, or neurologic dysfunctions [5]. Furthermore, this finding might be important evidence for preventing chemotherapy dose reduction because of physician bias.

There are some limitations in this study. First, the follow-up duration was short and the sample size was small. Second, this is a retrospective single center study. Third, there were no patients with peripheral vascular disease, dementia, DM with end organ damage, or AIDS. Despite these limitations, this study successfully applied the FCI and the CCI to newly diagnosed elderly multiple myeloma patients and revealed the superiority of FCI to CCI in predicting OS.

## 5. Conclusions

In this study, approximately 50% of elderly patients with newly diagnosed multiple myeloma had at least one comorbid disease at the time of diagnosis. Among host factors tested, performance status and a history of malignancy were the most important prognostic factors. The Freiburg comorbidity index is very simple to use and predicts overall survival better than the Charlson comorbidity index.

## Figures and Tables

**Table 1:** Definitions of the Charlson and Freiburg comorbidity indices.

Weight	Condition	Definition
*Charlson comorbidity index *		
1	Myocardial infarct	Hospitalization and electrocardiographic and/or enzyme change
	Congestive heart failure	Exertional or paroxysmal nocturnal dyspnea and responded symptomatically (or on physical examination) to digitalis, diuretics, or afterload reducing agents
	Peripheral vascular disease	Intermittent claudication or prior bypass for arterial insufficiency; gangrene or acute arterial insufficiency; untreated thoracic or abdominal aneurysm (≥6 cm)
	Cerebrovascular disease	Cerebrovascular accident with minor or no residual and transient ischemic attacks
	Dementia	Chronic cognitive deficit
	Chronic pulmonary disease	*Moderate*: dyspneic with slight activity, with or without treatment, and dyspneic with moderate activity despite treatment; *Severe*: dyspneic at rest, despite treatment, requires constant oxygen; CO_2_ retention and a baseline PO_2 _below 50 torr
	Connective tissue disease	SLE, PM, MCTD, polymyalgia rheumatic, and moderate to severe RA
	Ulcer disease	Required treatment for ulcer disease, including bleeding from ulcers
	Mild liver disease	Cirrhosis without portal hypertension or chronic hepatitis
	Diabetes	*Mild*: treated with insulin or oral hypoglycemics, but not with diet alone. *Moderate*: previous hospitalizations for ketoacidosis, hyperosmolar coma, or/and those with juvenile onset or brittle diabetics

2	Hemiplegia	Dense hemiplegia or paraplegia, as a result of either a cerebrovascular accident or other conditions
	Moderate or severe renal disease	*Severe*: on dialysis, had a transplant, and with uremia. *Moderate*: serum creatinine > 3 mg%
	Diabetes with end organ damage	*Severe*: with retinopathy, neuropathy, or nephropathy
	Any tumor	Solid tumors without documented metastases, but initially treated in the last 5 years
	Leukemia	AML, CML, ALL, CLL, and PV
	Lymphoma	HD, lymphosarcoma, WM, myeloma, and other lymphomas

3	Moderate or severe liver disease	*Severe*: cirrhosis, portal hypertension, and a history of variceal bleeding. *Moderate*: cirrhosis with portal hypertension, but without history of variceal bleeding

6	Metastatic solid tumor	Metastatic solid tumors
	AIDS	Define or probable AIDS (i.e., AIDS related complex)

*Freiburg comorbidity index *		
1	Renal impairment	eGFR_MDRD_ ≤ 30 mL/min/1.73 m^2^
	Performance status	Karnofsky performance status (KPS) score ≤ 70
	Moderate or severe lung disease	Same as CCI

Abbreviations: SLE, systemic lupus erythematous; PM, polymyositis; MCTD, mixed connective tissue disease; RA, rheumatoid arthritis; AML, acute myelogenous leukemia; CML, chronic myelogenous leukemia; ALL, acute lymphocytic leukemia; CLL, acute lymphocytic leukemia; PV, polycythemia vera; HD, Hodgkin disease; WM, Waldenstrom's macroglobulinemia; AIDS, acquired immune deficiency syndrome; eGFR, estimated glomerular filtration rate; MDRD, modification of diet in renal disease; CCI, Charlson comorbidity index.

**Table 2:** Baseline characteristics (*n* = 127).

Characteristics	*N* (%)	Median (range)
Age, years		71 (65–92)
65–74	93 (73.2%)	
≥75	34 (26.8%)	
Sex		
Male	62 (48.8%)	
Female	65 (51.2%)	
ECOG		
0-1	74 (58.3%)	
≥2	52 (40.9%)	
Unknown	1 (0.8%)	
Durie-Salmon stage		
1	10 (7.9%)	
2	28 (22.0%)	
3	89 (70.1%)	
International staging system		
1	23 (18.1%)	
2	51 (40.2%)	
3	47 (37.0%)	
Unknown	6 (4.7%)	
Lytic bone lesion		
Yes	112 (88.2%)	
No	14 (11.0%)	
Hemoglobin (g/dL)		9.66 (5.20–16.30)
<10	75 (59.1%)	
≥10	52 (40.9%)	
Platelets (×10^9^/L)		195 (44–484)
<100	11 (8.7%)	
≥100	117 (91.3%)	
Plasma cells in bone marrow (%)		43.88 (1.10–100)
≥40	58 (45.7%)	
<40	65 (51.2%)	
Serum calcium (mg/dL)		9.35 (7.00–15.70)
>11.5	11 (8.7%)	
≤11.5	115 (90.6%)	
Serum albumin (mg/dL)		3.28 (1.70–4.80)
≤3.5	85 (66.9%)	
>3.5	42 (33.1%)	
eGFR (mL/min/1.73 m^2^)		66.60 (5.90–170.5)
>30	112 (88.2%)	
≤30	15 (11.8%)	
Serum LD		
>UNL	27 (21.3%)	
≤UNL	74 (58.3%)	
Initial chemotherapy regimen		
Conventional (CP, MP, and others)	92 (78.0%)	
Novel agents (imid, bortezomib-based)	26 (22.0%)	
Treatment		
Chemotherapy	118 (92.9%)	
No chemotherapy	9 (7.1%)	

ECOG, eastern cooperative oncology group; eGFR, estimated glomerular filtration rate; LD, lactate dehydrogenase; UNL, upper normal limit; CP, cyclophosphamide and prednisolone; MP, melphalan and prednisolone.

**Table:** (a) Prevalence of comorbidities according to the Charlson comorbidity index.

Comorbidity	Yes	No
Myocardial infarct	7 (5.5%)	120 (94.5%)
Congestive heart failure	7 (5.5%)	120 (94.5%)
Peripheral vascular disease	0 (0%)	127 (100%)
Cerebrovascular disease	6 (4.7%)	121 (95.3%)
Dementia	0 (0%)	127 (100%)
Chronic lung disease	14 (11.0%)	113 (89.0%)
Connective tissue disease	2 (1.6%)	125 (98.4%)
Ulcer disease	8 (6.3%)	119 (93.7%)
Mild liver disease	2 (1.6%)	125 (98.4%)
DM	24 (18.9%)	103 (81.1%)
Hemiplegia	6 (4.7%)	121 (95.3%)
Moderate to severe renal disease	1 (0.8%)	126 (99.2%)
DM with end organ damage	0 (0%)	127 (100%)
Any tumor	8 (6.3%)	119 (93.7%)
Leukemia	0 (0%)	127 (100%)
Lymphoma	0 (0%)	127 (100%)
Moderate to severe liver disease	2 (1.6%)	125 (98.4%)
Metastatic solid tumor	2 (1.6%)	125 (98.4%)
AIDS	0 (0%)	127 (100%)

DM, diabetes mellitus; AIDS, acquired immune deficiency syndrome.

**Table:** (b) Prevalence of comorbidities according to the Freiburg comorbidity index.

Component	Yes	No
Renal impairment (eGFR ≤ 30 mL/min/1.73 m^2^)	15 (11.8%)	112 (88.2%)
Performance status (KPS ≤ 70)	52 (40.9%)	74 (58.3%)
Moderate or severe lung disease	14 (11.0%)	113 (89.0%)

eGFR, estimated glomerular filtration rate; KPS, Karnofsky performance status.

**Table:** (c) Patient distribution according to comorbidity indices.


(I) CCI total (comorbidity scores without age points)	
0	65 (51.2%)
1	37 (29.1%)
2	12 (9.4%)
3	5 (3.9%)
4	6 (4.7%)
6	1 (0.8%)
8	1 (0.8%)
(II) CCI total (comorbidity scores with age points)	
2	25 (19.7%)
3	49 (38.6%)
4	31 (24.4%)
5	9 (7.1%)
6	5 (3.9%)
7	6 (4.7%)
9	1 (0.8%)
13	1 (0.8%)
(III) CCI score group (with age points)	
Low (2-3)	53 (41.7%)
High (≥4)	74 (58.3%)
(IV) FCI	
0	59 (46.5%)
1	54 (425%)
2	12 (9.4%)
3	1 (0.8%)
Unknown	1 (0.8%)

CCI, Charlson comorbidity index; FCI, Freiburg comorbidity index.

**Table 4:** Univariate and multivariate Cox's regression analysis for overall survival.

	Univariate analysis			Multivariate analysis		
	*P* value	HR	95% CI	*P* value	HR	95% CI
ECOG	0.002	1.951	1.287–2.958	0.009	1.890	1.176–3.038
0-1						
≥2						
Chronic lung disease	0.028	1.941	1.073–3.510	0.300	1.425	0.730–2.778
Yes						
No						
eGFR (mL/min/1.73 m^2^)	0.012	2.139	1.183–3.869	0.228	1.515	0.771–2.976
>30						
≤30						
Any tumor	0.001	3.513	1.678–7.356	0.003	3.717	1.617–8.554
Yes						
No						
Metastatic solid tumor	<0.001	44.034	8.449–229.485	<0.001	85.847	14.628–503.822
Yes						
No						
Cerebrovascular disease	0.016	3.064	1.228–7.641	0.210	1.855	0.706–4.887
Yes						
No						
International staging system	0.024			0.250		
1						
2						
3						
Age, years	0.339	1.242	0.796–1.938			
65–74						
≥75						
Sex	0.692	0.920	0.610–1.388			
Male						
Female						
Durie-Salmon stage	0.242					
1						
2						
3						
Myocardial infarct	0.146	1.783	0.818–3.884			
Yes						
No						
Congestive heart failure	0.581	1.329	0.484–3.644			
Yes						
No						
Connective tissue disease	0.302	0.047	0.000–15.543			
Yes						
No						
Ulcer disease	0.196	0.571	0.244–1.334			
Yes						
No						
Mild liver disease	0.262	2.246	0.545–9.249			
Yes						
No						
DM	0.256	0.718	0.405–1.272			
Yes						
No						
Hemiplegia	0.081	0.353	0.110–1.135			
Yes						
No						
Moderate to severe renal disease	1.000	1.000	0.000–4.271*E*9			
Yes						
No						
Moderate-severe liver disease	0.302	2.100	0.514–8.583			
Yes						
No						
Initial chemotherapy regimen	0.844	1.058	0.603–1.858			
Conventional agents						
Novel agent						
CCI	0.061	0.677	0.450–1.018			
2-3						
≥4						
FCI	<0.001					
0						
1						
2-3						

HR, hazard ratio; CI, confidence interval; ECOG, eastern cooperative oncology group; eGFR, estimated glomerular filtration rate; DM, diabetes mellitus; CCI, Charlson comorbidity index; FCI, Freiburg comorbidity index.

**Table 5:** Serious adverse events (AEs).

	*N* (%)	
	Grade 0–3	Grade 4-5
Hematologic AE		
Anemia	103 (87.3%)	15 (12.7%)
Neutropenia	103 (87.3%)	15 (12.7%)
Thrombocytopenia	114 (92.2%)	4 (7.8%)
Febrile neutropenia	113 (90.8%)	5 (9.2%)

	Grade 0–2	Grade 3–5

Nonhematologic AE		
Infection	83 (70.0%)	35 (30.0%)
Diarrhea/constipation	105 (89.0%)	13 (11.0%)
Fatigue	117 (90.7%)	11 (9.3%)
Sensory neuropathy	108 (91.5%)	10 (8.5%)
Nausea/vomiting	111 (94.1%)	7 (5.9%)
Azotemia	112 (94.9%)	6 (5.1%)

Grade ≥4 hematologic AE	27 (22.9%)	
Grade ≥3 nonhematologic AE	59 (50.0%)	
Grade 5 AE	8 (6.8%)	
